# Green Synthesis of Silver Nanoparticles through Reduction with *Solanum xanthocarpum* L. Berry Extract: Characterization, Antimicrobial and Urease Inhibitory Activities against *Helicobacter pylori*

**DOI:** 10.3390/ijms13089923

**Published:** 2012-08-09

**Authors:** Muhammad Amin, Farooq Anwar, Muhammad Ramzan Saeed Ashraf Janjua, Muhammad Awais Iqbal, Umer Rashid

**Affiliations:** 1 Department of Chemistry, University of Sargodha, Sargodha 40100, Pakistan; E-Mails: makhan1111@yahoo.com (M.A.); dr_janjua2010@yahoo.com (M.R.S.A.J.); ma.chemist1002@gmail.com (M.A.I.); 2 Institute of Advanced Technology, Universiti Putra Malaysia, Serdang 43400, Selangor, Malaysia

**Keywords:** silver nanoparticles, *Solanum xanthocarpum*, anti-*Helicobacter pylori* activities, urease inhibitory activities, TEM, agar dilution method

## Abstract

A green synthesis route for the production of silver nanoparticles using methanol extract from *Solanum xanthocarpum* berry (SXE) is reported in the present investigation. Silver nanoparticles (AgNps), having a surface plasmon resonance (SPR) band centered at 406 nm, were synthesized by reacting SXE (as capping as well as reducing agent) with AgNO_3_ during a 25 min process at 45 °C. The synthesized AgNps were characterized using UV–Visible spectrophotometry, powdered X-ray diffraction, and transmission electron microscopy (TEM). The results showed that the time of reaction, temperature and volume ratio of SXE to AgNO_3_ could accelerate the reduction rate of Ag^+^ and affect the AgNps size and shape. The nanoparticles were found to be about 10 nm in size, mono-dispersed in nature, and spherical in shape. *In vitro* anti-*Helicobacter pylori* activity of synthesized AgNps was tested against 34 clinical isolates and two reference strains of *Helicobacter pylori* by the agar dilution method and compared with AgNO_3_ and four standard drugs, namely amoxicillin (AMX), clarithromycin (CLA), metronidazole (MNZ) and tetracycline (TET), being used in anti-*H. pylori* therapy. Typical AgNps sample (S1) effectively inhibited the growth of *H. pylori*, indicating a stronger anti-*H. pylori* activity than that of AgNO_3_ or MNZ, being almost equally potent to TET and less potent than AMX and CLA. AgNps under study were found to be equally efficient against the antibiotic-resistant and antibiotic-susceptible strains of *H. pylori*. Besides, in the *H. pylori* urease inhibitory assay, S1 also exhibited a significant inhibition. Lineweaver-Burk plots revealed that the mechanism of inhibition was noncompetitive.

## 1. Introduction

Preparation of nano-sized silver based materials, usually ranging in size from 1 to 100 nanometers (nm), is amongst the most emerging areas in the field of nanotechnology. Currently, the applications of nano materials is becoming increasingly important in order to address the problems associated with material sciences, including solar energy conversion, photonics [1], catalysis [2], microelectronics [3], antimicrobial functionalities [4], and water treatment [5].

A number of synthetic methods have been employed for the synthesis of silver-based nanoparticles involving physical, chemical [6] and biochemical techniques [7]. Chemical-based synthesis techniques are often discouraged as they involve the use of noxious reducing and/or stabilizing agents like sodium borohydride [8] and *N*,*N*-dimethylformamide [9] and toxic solvents [10].

With increasing focus on green chemistry, natural compounds like glucose [11], chitosan [12], soluble starch [13] and some microorganisms [14–17], *etc.*, have attracted considerable research interest as safer alternatives, reducing and stabilizing agents to synthesize the silver nanosphere. Synthesis of nanoparticles through biochemical routes, using plant extracts as reducing and capping agents, has received special attention among others, due to maintaining an aseptic environment during the process [18–24]. Therefore, medicinal plants having well established therapeutic importance are being widely used for the size- and shape-controlled synthesis of silver nanoparticles [25–28].

*Solanum xanthocarpum*, commonly known as yellow-berried nightshade, is a prickly plant, which grows wild in different regions of the Indo-Pakistan subcontinent. It has been reported that this plant contains several steroidal alkaloids like solanacarpine, solanacarpidine, solancarpine, solasonine, solamargine and other constituents, such as caffeic acid, coumarins (aesculetin and aesculin), steroids (carpesterol, diosgenin, campesterol, daucosterol) and triterpenes (cycloartanol and cycloartenol) [29]. The fruit from *S. xanthocarpum* has flavonoids quercitrin and apigenin glycosides as the major chemical constituents [29]. Various medicinal properties have been ascribed to different parts of this multipurpose herb. For example, the root is an expectorant, and is employed in folk medicine systems for the treatment of cough, asthma and chest pain as well as wound healing [29–33]. Fruits are edible, act as an anthelmintic, and are used as a remedy for the treatment of different ailments [32]. A recent study appraises the antihyperglycemic and antioxidant activities of leaf extracts from *S. xanthocarpum* on alloxan-induced diabetic rats [34].

It is now firmly established that gastric and duodenal ulcers are generally caused by *H. pylori* which survives and grows in acidic environments [35]. Triple therapy, including a proton pump inhibitor and any of the two antibiotics, such as AMX, CLA, MNZ and TET is frequently conducted for treating *H. pylori*-related infections [36]. Although an eradication rate of more than 80% has been reported by the use of relevant therapy, different side-effects including the emergence of antibiotic-resistant in *H. pylori* due to overuse of antibiotics are still to be addressed.

Therefore, there is a need to develop antimicrobial agents possessing enhanced efficacy against microorganisms and reduced toxicity for human cells. In this regard, many metals and their salts have been reported for possessing antibacterial activities against *H. pylori* [37,38]. The possible mechanism of action of metallic agents is the inactivation of *H. pylori* urease [39,40]. In this context, the appliance of silver nanoparticles (AgNps) in the field of medicine including wound dressings and medical devices, *etc.* is in practice [41]. However, an adequate assessment of the long-term effects of AgNps exposure on human physiology and their release into the environment is debatable. Most of the scientific literature on the toxicology of AgNPs has only been published in the past decade [42]. Many of these studies have revealed AgNPs to have mild toxicity against several cell lines as well as a number of aquatic organisms [43], and the mechanistic basis of these toxic effects is now an area of active research [44–46].

To the best of our knowledge, metal nanoparticles have not yet been tested against *H. pylori*. As a potential exists for the use of medicinal plant extracts as reducing and capping agents to prepare nanoparticles through green synthesis, so in the present work very stable silver nanoparticles (AgNps) were synthesized using *S. xanthocarpum* berry extract (SXE). SXE acted both as a reducing as well as a capping agent. Anti-*Helicobacter pylori* activities of synthesized AgNps were evaluated against 34 local isolates and two reference strains of *H. pylori* by the agar dilution method.

## 2. Results and Discussion

In the present work, AgNps have been synthesized by the reduction of aqueous silver ions using *S. xanthocarpum* berry extract. The effect of concentration of reacting substances, temperature, time and pH on synthesis rate, size and shape of the nanoparticles was studied.

### 2.1. UV-Visible Analysis of AgNps

The optical properties of AgNps were calculated by UV-Vis absorption spectroscopy, an important and most commonly used technique, to ascertain the formation stability of metal nanoparticles. Due to surface plasmon resonance (SPR), a strong absorption of electromagnetic waves is exhibited by metal nanoparticles in the visible range. The SPR phenomenon arises when nanoparticles are irradiated with visible light, because of the collective oscillations of the conduction electrons. It is well known that AgNps exhibit a yellowish-brown color in aqueous solution due to the excitation in UV-visible spectrum depending upon the particle size [47].

**Figure 1:**
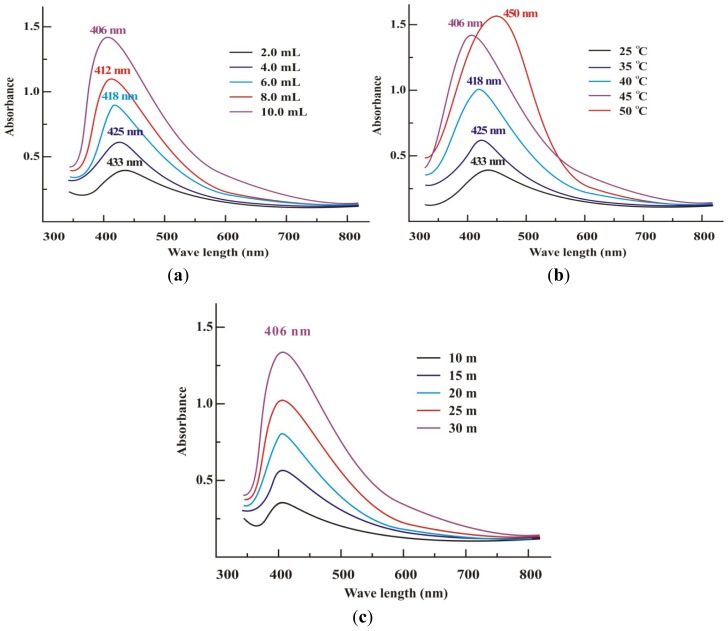

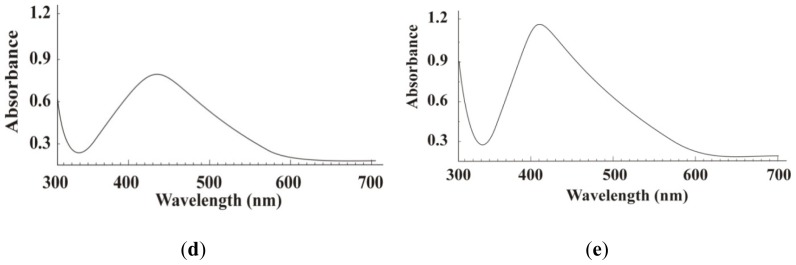
UV-visible spectra of typical sample S1 synthesized by the reaction of AgNO_3_ (10 mL, 1 mM) reacted with *S. xanthocarpum* berry extract (SXE) having concentrations from 2–10 mL at 45 °C for 30 min. (**a**) effect of SXE concentrations; (**b**) effect of temperature; (**c**) effect of reaction time; (**d**) effect of acidic conditions; (**e**) effect of basic conditions on the synthesis of S1.

The SPR bands in the UV–Vis spectra of AgNps colloidal suspension S1, S2, S3, S4 and S5 appeared at 406, 412, 428, 425 and 433 nm, respectively, Figure 1(a). For colloid S5 the SPR at 433 nm was broad. By increasing the concentration of SXE, the SPR bands for colloids S4, S3, S2 and S1 became sharper and for the typical sample S1 SPR appeared at 406 nm. The broad SPR at lower quantities of the SXE was due mainly to the formation of large anisotropic particles. This perhaps may occur because by lowering the quantity of SXE, the concentration of groups/molecules such as phenolics, alkaloids and sugars, *etc.* responsible for capping and stabilizing of nanoparticle reduces. Similar results have already been reported in the case of the stabilization effect of biological extracts on the formation of metal nanoparticles [14–15,22]. The increased bandwidth of colloid S5 is due to the fact that beyond a limit the bio-molecules in an extract cease to act as a capping agent. The fairly sharp and symmetrical SPR band observed for colloid S1 at 406 nm is indicative of small spherical nanoparticles.

Figure 1(b) shows the effect of temperature on the synthesis of typical sample S1. A broad peak of less intensity was observed at 433 nm for the colloidal suspension obtained after heating the reaction mixture at 25 °C for 2–3 h. With increase in temperature from 25 °C to 45 °C, the SPR peaks became sharper and sharper and for the yellowish brown solution, obtained after 30 min of stirring at 45 °C, an absorption band at 406 nm was obtained which suggested the formation of silver nanoparticles. It can be observed that an optimum temperature is required for the completion of reaction due to the instability of formed silver nanoparticles. The optimum temperature required for the completion of reaction was investigated to be 45 °C. Upon a further increase in temperature (up to 50 °C), a red-shift appeared from 406 nm to 450 nm due to increase in AgNps size. Further increase in temperature caused the broadening of the peak revealing the increased size of nanoparticles. This temperature dependent increase in the peak intensity showed the dependence of the silver ion reduction on the reaction temperature. It was observed that reduction rate of silver ions increased by increasing temperature. Similar results were reported by Pastoriza-Santos and Liz-Marzan [9]. This sharpness in absorbance peak depends on the size of the synthesized nanoparticle, as with higher temperature particle size may be smaller, which results in sharpness of the plasmon resonance band of AgNPs [48]. Initially, the size was reduced due to the reduction in aggregation of the growing nanoparticles. Increasing the temperature beyond 45 °C aids the growth of the crystal around the nucleus resulting in a decrease in absorption.

Effect of time on the completion of reaction was studied by UV-Vis spectroscopy for colloidal suspension S1. An absorption band of very low intensity appeared at 406 nm for the colloid after 10 min of stirring, Figure 1(c), which changed into a clearly visible peak after 30 min at the same absorbance, indicating the presence of spherical AgNps. The absorption peak intensity increased rapidly with increase in reaction time from 10 to 30 min due to the continuous formation of AgNps in the reaction system. It was, therefore, observed that an optimum time is required for the completion of reaction due to the instability of formed silver nanoparticles. The optimum time required for the completion of reaction was recorded to be 30 min.

pH is another important factor affecting the reduction of silver ions. The effect of pH on the reduction of silver ions was studied by UV-Vis spectroscopy and is shown in Figure 1(d,e). At pH 4.0, no absorption peak was observed in the range of 400–450 nm for the colloidal suspension of all samples even after 24 h of the reaction. However, an absorption band appeared at about 440 nm when pH increased from 4 to 5 indicating the formation of AgNps, Figure 1(d). It was observed that the absorption peak intensity increased gradually with an increase in pH, suggesting that the reduction rate of silver ions increases with an increase in pH. The formation of AgNps was suppressed by acidic conditions and enhanced by basic conditions. At lower pH (pH 5), larger nanoparticles were formed, whereas, at higher pH (pH 9), smaller and highly dispersed nanoparticles formed, Figure 1(e).

### 2.2. PXRD and TEM Studies

**Figure 2:**
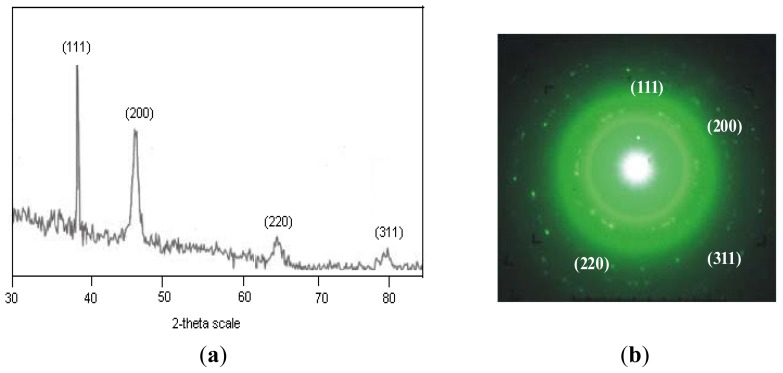

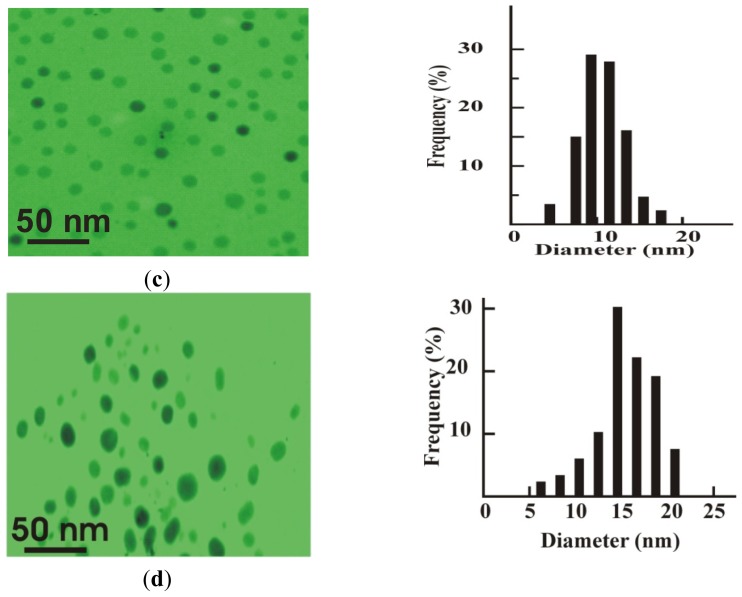
(**a**) XRD pattern of silver nanoparticles S1; (**b**) SAED of S1; (**c**) TEM image and size distribution histogram of S1; (**d**) TEM image and size distribution histogram of S2.

The XRD pattern of dried silver nanoparticles S1 is shown in Figure 2(a). The four diffraction peaks at 38.2°, 44.1°, 64.3° and 78° are indexed as (111), (200), (220) and (311) planes of face centered cubic silver. The data obtained was matched with the database of Joint Committee on Powder Diffraction Standards (JCPDS) file No. 04-0783. The (200), (220) and (311) Bragg reflections are weak and broadened relative to the intense (111) reflection. This feature indicates that the nanocrystals are (111)-oriented [49] as confirmed by TEM measurements. The crystalline nature of S1 was further evidenced by the selected area electron diffraction (SAED) pattern Figure 2(b) with bright circular spots corresponding to (111), (200), (220) and (311) Bragg reflection planes [50]. This indicates that the prepared silver nanoparticles may be enriched in (111) facets and thus the (111) plane seems to be preferentially oriented parallel to the surface of the supporting substrate. The average particle size of AgNps was calculated by the use of full width at half maximum (FWHM) of *face*-*centered cubic* (111) using the Debye–Scherrer equation, *K λ*/*βcosθ*, where K is the Scherrer constant with value from 0.9 to 1, λ is the wavelength of the X-ray, β is the full width at half maximum and θ is the Bragg angle in radians. From the Scherrer equation the average crystallite size of silver nanoparticles S1 and S2 was found to be about 10 nm and 15 nm, respectively.

Typical TEM images of colloid S1 and S2 with size distribution histograms are shown in Figure 2(c,d), respectively. It is evident from the Figure 2(c) that the morphology of S1 is almost spherical, which is in agreement with the shape of the SPR band in the UV–Vis spectra. The average particle size calculated for S1 was 10 nm with size ranging from 4 to 18 nm. Whereas the average particle size for sample S2 was calculated as 14 nm with an average size ranging from 7 to 18 nm. These results are in accordance with the shape of the SPR band and with the particle size calculated from XRD analysis.

### 2.3. *In Vivo* Anti-*H. pylori* Activities

MIC_90_ values for a typical AgNp sample named as S1, silver nitrate and the standard drugs are listed in Table 1. The sample S1 exhibited stronger anti-*H. pylori* activity (MICs 2–8 μg mL^−1^) than silver nitrate (MIC 16–64 μg mL^−1^), TET (MIC 0.25–64 μg mL^−1^), and MNZ (2–512 μg mL^−1^), however it was found to be less potent than AMX (0.125–4 μg mL^−1^) and CLT (0.125–8 μg mL^−1^). The antibacterial activity of S1 against AMX-resistant, CLT-resistant, TET-resistant and MNZ-resistant clinical isolates was nearly comparable to those against AMX, CLT, TET, and MNZ susceptible isolates (Table 1). Furthermore, S1 was also found to be equally effective against the strains showing double (SA-1, SA-6, SA-7, SA-21, SA-28, SA-31 and SA-34) and triple (SA-3, SA-5 and SA-17) drug resistance. For some cases (SA-6, SA-31, MICs at 4 μg mL^−1^) the isolate exhibiting a strong resistance to AMX was found to be susceptible to S1 with MIC at 2 μg mL^−1^. This trend was frequently observed in the case of other standard drugs.

### 2.4. Urease Inhibitory Assay

Urease inhibitory activity of silver nanoparticles S1 has been studied by the phenol red method. The results are shown in Table 2 in the form of percent inhibition. It was found that urease inhibitory activity increased linearly with increased concentration of S1. Under the conditions used in the present investigation, *H. pylori* urease inhibition by S1 followed Michaelis-Menten kinetics. Kinetic studies of the enzyme in the presence of S1 were presented by Lineweaver-Burk plots in which a reciprocal of enzyme activity was taken on the Y-axis and a reciprocal of substrate concentration on the X-axis. The trend of lines of different concentrations of the inhibitor gave the idea about inhibition.

**Figure 3:**
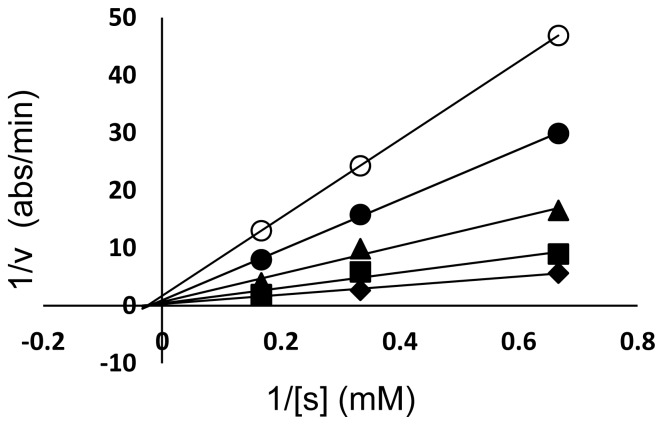
Inhibition of *H. pylori* urease S1 Lineweaver–Burk plots of the reciprocal of initial velocities *vs.* reciprocal of four fixed substrate concentrations in absence (○); presence of 80 mM (●); 60 mM (▲); 40 mM (■); 20 mM (◆). Lineweaver-Burk graphs.

Line weaver-Burk plots indicated that S1 is a non-competitive type of inhibitor. The plot of 1/V_o_ *vs.* 1/[s] (Figure 3) consisted of a family of straight lines that intersected each other on the same point on X-axis depicting the same K_m_ values with varying values of V_max_ with the concentration of the inhibitor. Furthermore, it was also clear from the graphs that inhibition increased almost linearly. Therefore, it may be suggested that the mechanism was non-competitive in which the inhibitor and substrate were both attached to the enzyme non-competitively [51].

Metal based nano-preparations have gained importance due to a variety of their applications in various fields [1–10]. The synthesis techniques involve the reduction of the corresponding metallic ion by a reducing agent. The newly synthesized nanoparticles are highly unstable and become aggregated in the absence of a suitable capping agent. Chemical techniques involving the use of chemicals and solvents are described as toxic and carcinogens, and have been suggested to be eliminated by the Conferences on Harmonization [10]. It is, therefore, necessary that the nanoparticles must be synthesized using such techniques which being benign to the environment must be cost-effective. The present investigation explored the use of fruit from an important medicinal plant *S. xanthocarpum* [31–34] as a template for the synthesis of AgNps by acting both as a reducing and a capping agent. The methanolic extract was found as an efficient capping as well as a stabilizing agent, whereas aqueous and acetone extracts did not exhibit good results (data not shown). The possible reason for this may be the dissolved phyto-chemicals present in the methanolic extract. During synthesis, it was found that an increase in concentration of *S. xanthocarpum* berry extract affected the morphology of the silver nanoparticles and the conditions were maintained such that finally 10 mL of the extract added to 20 mL of 1 mM silver nitrate solution resulted in a color change to golden brown, which is indicative of the formation of silver nanoparticles [16]. It has been established [52] that the optical absorption spectra of metal nanoparticles are dominated by SPR, which shift to longer wavelengths with increasing particle size. Therefore, a SPR band shift from 406 nm to 433nm (S1–S5) is indicative of the fact that an optimum concentration (10 mL) of extract is required for the synthesis of AgNps. Also, the shape of the SPR band of AgNps is strongly dependent on the particle size [53,54]. It has already been demonstrated [22] that only a single SPR band is expected in the absorption spectra of spherical nanoparticles, whereas anisotropic particles could give rise to two or more SPR bands depending on the shape of the particles. Furthermore, the number of SPR peaks increases as the symmetry of the nanoparticle decreases [54]. Therefore, the single symmetrical SPR band at 406 nm confirms the spherical morphology of the typical sample S1. *S. xanthocarpum* berries have been reported to contain several steroidal alkaloids like solanacarpine, solanacarpidine, solancarpine, solasonine, solamargine and other constituents like caffeic acid, coumarins like aesculetin and aesculin, steroids carpesterol, diosgenin, campesterol, daucosterol and triterpenes like cycloartanol and cycloartenol [55]. The flavonoids quercitrin and apigenin glycosides are the major chemical constituents present in *S. xanthocarpum* fruit [30]. Carbohydrates, flavonoids, terpenoids and certain proteins present in SXE may have a reducing and stabilizing effect in the nanoparticle synthesis. The synthesized AgNps were tested for their anti-*H. pylori* activity by the use of agar dilution method and their MICs were compared with silver nitrate and some of the standard drugs being used in anti-*H. pylori* therapy. AgNps exhibited more potent bactericidal activity than ionic silver (silver nitrate) and some of the standard drugs. The reason for much lower MICs and stronger antibacterial activities of S_1_ than AgNO_3_ might be due to smaller size and the presence of (111) lattice plane in the former [56]. Metal atoms in nano size provide a significantly large surface area in contact with the bacterial effluent. Considering a hypothetical case with spherical particles of uniform size, a reduction in the particle size from 10 μm to 10 nm would increase the contact surface area by 10^9^. Such a large contact surface is expected to enhance the extent of bacterial elimination. The size-dependent bactericidal activity of silver nanoparticles against gram-negative bacteria has already been reported [57,58]. Literature reports reveal the bactericidal activity of silver nanoparticles of either a simple or composite nature [59,60]. Multiple investigations have been performed to show the antibacterial activity of metals and metals chelated with some ligands against *H. pylori* [61].

To the best of our understanding, the present investigation can be considered to be the first of its kind reporting the anti-*H. pylori* activity of silver nanoparticles and their comparison with silver ions and some standard antibiotics being used in triple and quadruple therapy for the eradication of *H. pylori*. No standard susceptibility testing methods have yet been recommended for nanoparticles and metallic ions. Although broth dilution test and agar dilution test are both standard *in vitro* susceptibility tests recommended by the CLSI, due to some advantages, the agar dilution method has been adopted in the present investigation [59]. The exact mechanism of anti-*H. pylori* activity of silver nanoparticles is not clearly understood. However, there may be two possible explanations for this mechanism. In this direction, one theory speculates that a very small quantity of silver ions may enter into the bacteria to inhibit the microorganism’s respiratory system, electron transport system and enzymes, whilst another theory believes that silver ions may directly interfere with nickel in *H. pylori* urease, thereby making the enzyme inactive [59,62,63]. Urease, in *H. pylori*, converts urea into ammonia, which then counters the stomach acid, thereby creating a neutralizing environment for protecting *H. pylori* from the acid in the stomach. Gastric infection with *H. pylori* may lead to the onset of various gastric-related diseases. Most patients specifically with duodenal ulcer can be cured by killing *H. pylori* with antibiotics and proton pump inhibitors [35]. Several triple- or quadruple-antibiotic therapies with proton pump inhibitors have been shown to be effective in the eradication of *H. pylori* [36], but no single treatment regime is considered the final treatment of choice. Therefore, the specific inhibition of urease activity has been proposed as a possible strategy to inhibit *H. pylori*. [37,38].

Hence, there is a dire need for the eradication of *H. pylori* by single-therapy if possible. Bismuth is the only metal being widely used as antibacterial agent against *H. pylori* as a part of triple therapy. Due to potential toxic effects of bismuth, some other metals [37,38] including silver, having well defined antimicrobial activities, can be explored as possible treatments for gastrointestinal symptoms and infections. Silver compounds have been reported as anti-ulcer agents in animals and man, and their role in wound healing is well established [64]. Since *H. pylori* are a major peptic-ulcer causing agent, it raised the possibility that a part of the anti-ulcer activity of silver nanoparticles could be due to its effects on the growth of *H. pylori*.

## 3. Experimental Section

### 3.1. Materials/Chemicals/Reagents and Strains

Fully ripened *S. xanthocarpum* berries were collected from Soon Valley of the Punjab province of Pakistan in the month of June 2010 and brought to the Department of Biological Sciences, University of Sargodha, Sargodha, Pakistan. The specimens were further identified and authenticated by Amin Ullah Shah (Assistant Professor, Department of Biological Sciences, University of Sargodha (UoS), Sargodha, Pakistan). A specimen of the berries was also submitted to the herbarium of the UoS. The berries were dried under shade for 4–5 weeks.

Silver nitrate (AgNO_3_, analytical grade) was purchased from Sigma–Aldrich. A total of 34 clinical isolates of *H. pylori* have already been isolated and characterized by the same group and published elsewhere [37]. Two reference strains of *H. pylori* NCTC 11637 and NCTC 11638 were purchased from the Health Protection Agency, London, UK.

### 3.2. Isolation of S. xanthocarpum Berry Extract (SXE)

Dried berries (50 g) were ground using an ordinary coffee grinder and extracted with methanol for 24 h using a conventional Orbital Shaker. After filtration, the residue was re-extracted with fresh solvent; both the extracts were pooled and then concentrated to dryness with a rotary evaporator (45 °C). The crude concentrated extract was quantitatively diluted with methanol and preserved at −4 °C for further experiments.

### 3.3. Synthesis of Silver Nanoparticles

Silver nitrate (20 mL; 1 mM) was added dropwise into SXE (10 mL) while stirring and heated (45 °C) in a water bath at pH 9. The resulting solution became yellowish brown after 30 min of heating, indicating the formation of silver nanoparticles [8]. The colloidal suspension thus obtained was centrifuged at 4000 rpm for 30 min and the pellet obtained after discarding the supernatant was re-dispersed in deionized water. The centrifugation process was repeated 2 to 3 times for the removal of any adsorbed substances on the surface of silver nanoparticles (AgNps). The synthesized nanoparticles were lyophilized and recovered in powdered form using a Heto-Holten A/S, DK-3450 freeze dryer (Allerd, Denmark). Different samples of AgNps were synthesized by varying the concentration of SXE and silver nitrate solution as presented in Table 3.

### 3.4. Factors Affecting Synthesis Rate, Size and Shape of Silver Nanoparticles

#### 3.4.1. Temperature Effect

To study the effect of temperature on the synthesis of AgNps, a typical sample, namely S_1_, was synthesized at 25 °C, 35 °C, 40 °C, 45 °C and 50 °C. Electronic absorption spectra of the aqueous colloidal suspensions were recorded at each temperature range.

#### 3.4.2. Time Effect

To study the effect of time on the completion of reaction, the reaction was monitored from 0 to 30 min and aliquots were taken after every 5 min time interval for S_1_. The absorbance of the resulting solutions was recorded.

#### 3.4.3. pH Effect

pH of the reaction mixture was maintained at 4, 7 and 9, respectively, by using 0.1 N HCl and 0.1 N NaOH. The absorbance of the resulting solutions was measured spectrophotometrically.

#### 3.4.4. Effect of Volume of SXE Extracts

Effect of SXE to AgNO_3_ volume ratio on the shape and size of AgNps was studied by varying the volume ratio of AgNO_3_ and SXE. The AgNps samples thus synthesized are presented in Table 3. The absorbance of the resulting colloids was recorded.

### 3.5. Characterization Techniques

#### 3.5.1. UV-Vis Spectroscopy

The UV-Vis absorption spectra of the AgNps were recorded using Pharmaspec UV-1700 (Shimadzu Corporation, Tokyo, Japan) UV-Visible spectrophotometer at room temperature. The scanning range for the samples was 300–800 nm with a resolution of 1 nm at a scan speed of 300 nm/min.

#### 3.5.2. Transmission Electron Microscopy (TEM)

TEM measurements were performed on JEM-1200EX electron microscope (JEOL, Tokyo, Japan) instrument by operating at an accelerating voltage of 120 kV. The size distribution was obtained by measuring the diameter of more than 135 particles and using Origin 7.5 software (Origen Lab Corporation, Northampton, Massachusetts*,* USA), and confirmed by making the calculations by the use of Debye-Scherrer equation (D = Kλ/βcosθ) from the highest intensity of XRD pattern.

#### 3.5.3. X-ray Diffraction Analysis

The X-ray diffraction analysis was conducted on Bruker D8 Discover Powder Diffractometer (Berlin, Germany) using monochromatic Cu K α radiation (θ = 1.5406 Å) operating at a voltage of at 40 kV and a current of 30 mA at room temperature. The intensity data for the lyophilized nanosilver powder were collected over a 2θ range of 10°–80°. To obtain information about sub-structure and topography of the nanoparticles, AFM measurements were carried out in a Scanning Probe Microscope SPM-9500 J3 (Shimadzu Corporation, Tokyo, Japan) under normal atmospheric conditions. The sample was observed within an area of 5.0 × 5.0 μm using contact mode. A freshly prepared sample was deposited on fine metal surfaces, air dried in a dust-free environment and the resultant smooth surfaces were subjected to AFM analysis.

### 3.6. *In Vitro* Anti-*Helicobacter pylori* Activity

*In vitro* anti-*H. pylori* activity of the synthesized AgNps was determined by the agar dilution method in accordance with the guidelines provided by CLSI [65]. The results were recorded as minimum inhibitory concentration (MICs). Different concentrations of the synthesized AgNps were prepared by the sample digestion method. Silver nanoparticles were centrifuged and the pellet thus obtained was washed several times with deionized water in order to remove un-reacted silver ions. The removal of un-reacted silver was further confirmed by treating each filtrate with 1 mM HCl until no silver chloride was precipitated. In order to determine the concentration of AgNps for susceptibility studies, the pellet free from silver ions was divided into two halves. One half was dissolved in aqua-regia and the solution evaporated near to dryness. The process of dilution and dryness was repeated three times. In this way, the AgNps were converted to AgNO_3_. The concentration of silver ions was determined by atomic absorption spectrometry. The other half of the nanoparticles, having a concentration almost equal to the first half, was used for anti-*H. pylori* activities. Fourteen wells on a 96-wells plate were filled with a two-fold serially diluted colloidal suspension of silver nanoparticles in distilled water having final concentrations of 128 to 0.125 μg mL^−1^. The control was filled with distilled water only. The final concentrations of other test compounds (AMX, TET, CLA) were also adjusted from 128 to 0.125 μg mL^−1^ by dissolving in DMSO. The control was filled with DMSO only. These dilutions were transferred to molten Muller Hinton infusion agar plates.

The frozen clinical isolates were thawed, diluted using the Muller Hinton infusion broth (MHIB), and adjusted to 10^7^ CFU mL^−1^. Culture (5 mL) was streaked over the agar plates. The plates were incubated under microaerophillic conditions in sealed bags at 37 °C for 72 or 96 h as appropriate. The MIC_90_ were determined as per standard procedure. The breakpoints to define a resistant strain according to Megraud *et al.*, [36] were found to be clarithromycin 1 μg mL^−1^; and according to Wu *et al.*, [66] 0.5 μg mL^−1^ and 16 μg mL^−1^ for amoxicillin and tetracycline, respectively. Each experiment was performed as triplicate.

### 3.7. Urease Inhibition Assay and Kinetics

#### 3.7.1. Isolation of *H. Pylori* Urease

*H. pylori* (NCTC-11638, Health Protection Agency, London) was grown in MHIB, supplemented with 10% fetal bovine serum for 24 h at 37 °C under microaerobic conditions (Campygen Oxford, UK). The Mao method [67] of *H. pylori* urease isolation has been followed. Briefly, the broth cultures (50 mL, 2.0 × 10^8^ CFU mL^−1^) were centrifuged (5000× *g*, 4 °C) and the collected bacterial mass after being washed twice with phosphate-buffered saline (pH 7.4) was stored at −80 °C. Afterwards, *H. pylori* was thawed to room temperature, and mixed with 3 mL of distilled water, protease inhibitors and sonicated for 60 s. Following centrifugation (15,000 *g*, 4 °C), the supernatant was desalted through SephadexG-25 column (Pharmacia Bio-tech, Uppsala, Sweden). The resultant crude urease solution was added to an equal volume of glycerol and stored at −4 °C for further use.

#### 3.7.2. Urease Inhibitory Assay

The reaction mixture consisting of phosphate buffer solution (55 μL, 3 mM, 4.5 pH), 25 μL of urease enzyme solution and test compound (5 μL, of S1) was incubated at 30 °C for 15 min in 96 well plates. The urease activity was determined by measuring the ammonia production using the indophenol method, as described by Weatherburn [68]. Briefly, 45 μL of each phenol reagent (1% (w/v) phenol and 0.005% (w/v) sodium nitroprusside) and 70 μL of alkali reagent (0.5% (w/v) NaOH and 0.1% active chloride, NaOCl) were added to each well. The increasing absorbance at 630 nm was measured after 50 min, using a micro-plate reader. The results were processed using the in-built software of the microplate reader. Percent inhibitions were measured by the formula 100–(OD)_test well_/(OD)_control_. Thiourea was used as control. IC _50_ values of the concentrations that inhibited the hydrolysis of the substrate were calculated using the EZ-Fit kinetic program (Perrella Scientific Inc., Amherst, Massachusetts, USA). The Lineweaver–Burk plots were drawn and the values of Michaelis-Menten constant K_i_ were calculated from the slopes of each line in the plot. Four concentrations of substrate (urea; 80 mM, 60 mM, 40 mM, 20 mM) were set for each sample. Each experiment was performed as triplicate.

## 4. Conclusions

A green method has been reported for the synthesis of silver nanoparticles using *S. xanthocarpum* berry extract as a reducing and capping agent. It was found that high pH value, temperature and molar ratio of *S. xanthocarpum* extract (SXE) to AgNO_3_ could accelerate the reduction rate of Ag^+^ and affect the Ag nanoparticle size. The adopted method is compatible with green chemistry principles as the SXE serves as a matrix for both reduction and stabilization of the silver nanoparticles synthesized. At a given SXE concentration, the efficiency of nanoparticle synthesis increases with silver nitrate concentration and reaction time, a property attributable to the large reduction capacity of the SXE. The synthesized Ag nanomaterials exhibited anti-*H. pylori* and urease inhibitory activities. The anti-*H. pylori* activity of AgNps was compared with silver nitrate. The work suggests that silver nanoparticles may provide potential applications as antibacterial and urease inhibitory agents to eradicate *H. pylori*-related infections.

## Figures and Tables

**Table 1:** Minimum inhibitory concentrations (MIC_90_ μg mL^−1^) of S1, AgNO_3,_ AMX, CLT, MNZ and TET against 34 clinical isolates and 2 reference strains of *H. Pylori*.

*H. pylori* strains	AMX	CLT	TET	MNZ	AgNp (S1)	AgNO_3_
Reference strains						
NCTC-11637	0.5	0.5	2	16	4	16
NCTC-11638	0.125	0.5	2	4	2	16

Clinical isolates						
SA-1	0.125	0.25	16	32	4	16
SA-2	0.25	0.25	1	4	2	16
SA-3	0.125	1	32	64	4	32
SA-4	0.125	0.25	1	2	4	32
SA-5	0.25	4	32	16	8	64
SA-6	4	0.5	2	16	2	64
SA-7	2	0.25	4	32	4	32
SA-8	0.25	0.25	1	64	2	64
SA-9	0.125	0.5	0.5	128	2	64
SA-10	0.25	0.25	1	256	2	64
SA-11	0.25	0.25	0.25	32	2	32
SA-12	0.125	0.5	0.25	64	2	32
SA-13	0.5	0.125	2	16	2	32
SA-14	0.5	0.5	64	4	2	32
SA-15	0.25	0.125	8	256	4	32
SA-16	0.25	0.5	8	512	2	32
SA-17	1	8	8	512	2	32
SA-18	0.125	0.125	0.5	32	4	32
SA-19	0.25	0.125	0.25	32	8	32
SA-20	0.5	0.125	0.5	32	4	16
SA-21	1	0.5	32	64	4	32
SA-22	0.25	0.125	0.25	16	4	16
SA-23	0.25	0.125	0.5	8	4	64
SA-24	0.25	0.5	0.25	8	4	64
SA-25	0.25	0.125	0.5	8	2	64
SA-26	0.25	0.5	0.25	64	2	32
SA-27	0.5	0.125	0.25	64	4	32
SA-28	0.5	8	0.25	512	2	16
SA-29	0.125	0.5	0.25	512	2	64
SA-30	0.5	0.125	1	512	2	64
SA-31	4	0.5	1	256	2	32
SA-32	0.125	0.125	0.25	16	2	16
SA-33	0.125	0.125	1	16	2	32
SA-34	2	0.5	0.25	16	2	32

**Table 2:** Inhibition (%) of *H. pylori* urease by S1.

AgNp Sample	Inhibition at 16 μM	Inhibition at 8 μM	Inhibition at 4 μM	Inhibition at 2 μM	Inhibition at 1 μM
S1	64.00 ± 1.06	32.13 ± 1.12	16.10 ± 0.52	8.86 ± 1.56	1.34 ± 1.10

The values are mean ± SD of triplicate measurements.

**Table 3:** Synthesized silver nanoparticles with varying concentrations of SXE.

AgNps sample	Volume of SXE (mL)	Volume of AgNO_3_ (mL)
S_1_	10	20
S_2_	08	20
S_3_	06	20
S_4_	04	20
S_5_	02	20
